# Mean-Field Games for Marriage

**DOI:** 10.1371/journal.pone.0094933

**Published:** 2014-05-07

**Authors:** Dario Bauso, Ben Mansour Dia, Boualem Djehiche, Hamidou Tembine, Raul Tempone

**Affiliations:** 1 Dipartimento di Ingegneria Chimica, Gestionale, Informatica, Meccanica, Palermo, Italy; 2 SRI Center for Uncertainty Quantification in Computational Science and Engineering, King Abdullah University of Science and Technology, Thuwal, Kingdom of Saudi Arabia; 3 Department of Mathematics, Royal Institute of Technology (KTH), Stockholm, Sweden; University of Westminster, United Kingdom

## Abstract

This article examines mean-field games for marriage. The results support the argument that optimizing the long-term well-being through effort and social feeling state distribution (mean-field) will help to stabilize marriage. However, if the cost of effort is very high, the couple fluctuates in a bad feeling state or the marriage breaks down. We then examine the influence of society on a couple using mean-field sentimental games. We show that, in mean-field equilibrium, the optimal effort is always higher than the one-shot optimal effort. We illustrate numerically the influence of the couple’s network on their feeling states and their well-being.

## Introduction

We look at marital interactions and relationships. Here relationship refers to the unit (couple), rather than to the two persons. We focus on a very ephemeral thing: what happens to the couple as they interact over time. It is not either person, it is something that happens when they are together, i.e., in a couple. The couple will create something that we call a “feeling state” as they talk to each other, as they smile, as they move. We model the feeling state of a couple as a noisy differential equation influenced by random events, parents, children, friends and the social distribution of marital status (mean-field of states), where the term “mean-field” refers to the distribution of couple feeling states in the society.

**Why a mathematical model for marital interaction?** Putting the study of social relationships on mathematical footing represents a major advance in our ability to understand and perhaps regulate these relationships for the betterment of all mankind [3]. The novelty of the present study inheres in the analysis of the social distribution of feeling states (mean-field) on a generic couple.

**On the influence of couple-feeling-states in society :** Since the society, friends and parents of a couple may influence them in an aggregative manner, a mean-field approach is suitable for such an interaction. Here the distribution of states (mean-field)- of all couples in the society plays an important role in the payoff and in the effort used to maintain a marriage. Game theory is a branch of mathematics which studies strategic interactions. Our idea is to use mean-field games to capture the influence of society distribution of states on a generic couple.

In their pioneering work in [3], Gottman et al. have widely illustrated the importance of mathematical theory in the field of marital research. In particular, they have shown that mathematical models help us to better understand the sentimental dynamics and hence to propose an appropriate intervention or therapy for a couple. The authors have developed a model to analyze marital instability, by calibrating a system of difference equations for the evolution of partners’ emotions during a conversation. The interaction dynamics is described by a function of experience and whose intuitive understanding of the influence among partners during conflict. More recently, Rey [4] formulated the sentimental relationship of a couple as an optimal control problem. A state variable monitors the wellness of the relationship whose natural decay in time must be counteracted with effort according to a widely accepted principle in marital psychology. Stationary sentimental steady states are examined in [4], [5] the context of infinite horizon discounted optimal control problems.

None of these previous works considered the influence of model uncertainty, noise, random events, and the social distribution of states (mean field). In this work we examine these important issues.

This paper has three main features:

- We introduce stochastic optimal control in marital interaction. We explicitly characterize the optimality equations under random fluctuations which comes from model uncertainty in the drift function of the feeling state.
- We examine the impact of mean-field and effort in the feeling state of the couple.
- We propose a mean-field game model for marriage in a non-linear setup where generically two stable states (divorce and marriage) are observed. We study the impact of the network of couples on the feeling state. We show that, in mean-field equilibrium, the optimal effort of the mean-field sentimental game is always higher than the one-shot optimal effort if the social contribution is controlled.

While the study here focuses on marital interaction, the mathematical methodology can be applied to other situations such as a society’s ecological or smoking behavior. To the best our knowledge this is the first attempt to model and understand marital interactions through mean-field influence and stochastic games.

The remainder of the paper is organized as follows. In the next section we present a generic model of sentimental dynamics examining both controlled and uncontrolled situations. After that we introduce mean-field sentimental games. Then, we analyze the optimal behavior and mean-field sentimental equilibria with and without noise. As an illustration we examine an interesting sentimental dynamics wherein marriage and divorce can be seen as “stable” steady states. Adding strategic effort behavior into such couple dynamics can change the long-term behavior of their feeling state if the system is uncontrolled.

## Model and Methods

We first present a basic mathematical framework for feeling state and well-being of a couple. We will explain below how the model can be modified to include the (positive or negative) impact of the society (and the “social pressure”) on a generic couple. We describe the sentimental dynamics, define the payoff functional for the marital interaction and state the optimal control problem.

### Governing Feeling State Equation

The feeling state (feeling level) *x* of the couple is modeled as an Itô’s stochastic differential equation:
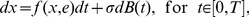
(1)where 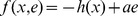, 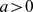 represents the expected variation of the feeling in a short time window, 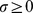, 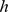 is smooth and goes to infinity with 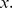 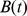 is a standard Brownian motion. The effort function 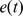 can be controlled by the couple. The sentimental dynamics is expected to start at a high feeling level 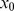 greater than 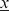 where 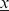 is a certain threshold value below which the relationship of the couple is not considered as satisfactory. The parameter 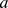 represents the efficiency of the effort.

**Remark 1** *The deterministic part of the governing feeling state is an experimental derivation from marital studies* [*3*]–[*5*] *. However, here, the function 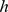 is not necessarily monotone in 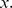 We introduce a stochastic term into the sentimental dynamics of the existing literature* [*3*]–[*5*] *for multiple reasons: (1) shocks, (2) random events in the social network of the couple may affect the couple, (3) the drift funtion 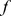 may not be perfectly known. We add an uncertainty term into it.*

**Remark 2** *If the marriage starts with a feeling level 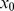 and is stopped at the first time that 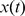 is below a certain threshold 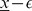 or one of the couple member dies (life expectancy is set to 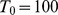 years) then the length of the horizon is expected to be finite. This is why we consider a finite horizon problem. Let 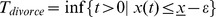, one can take 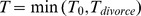 which means that 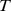 is a random variable.*

### Setting of the Problem

Based on the feeling state and the effort, we define the payoff functional for a couple during 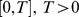 as
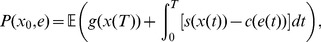
(2)where 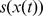 represents the well-being of the couple at state 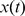 and 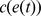 is the cost associated to the effort 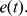 The instantaneous payoff captures the risk at time 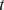 but we have limited our analysis to the long-term risk-neutral setup. More details on risk-sensitive mean-field type control and games can be found in [8], [9].

We define a couple as a union of two adult persons having certain emotional and physical interactions, and living together. We define family as the direct ascendants and the direct descendants of the conjoint. In this sense, we read the quantity 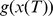 as the heritage that the couple bequeaths to the family in terms of well-being. This heritage will influence the next generation sentimental game. It is reasonable to assume that the function 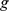 increases with the feeling state and 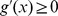.

The function 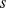 is the well-being function of the couple. It depends on the feeling state 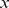 in the sense that, the higher the feeling state, the more joyful the couple. The implicit purpose of the control problem is to increase well-being while taking into consideration the cost. Thispurpose achieved by expanding the feeling state above a certain satisfactory level.

Contrastingly, couples with low feeling state are more or less happy and have the tendency to seize again. This tendency is expressed by taking actions; caring for children, giving gifts, cooking special meals, making concessions, in short, handling the couple according to his or her expectation. We define the effort as a constructive action from one conjoint to the other conjoint. It might be useful for the reader to notice that our definition of effort does not take into account counterproductive actions. For example, knowing that Juliette does not like football, it is negative that Romeo takes her to the Camp Nou Stadium for FC Barcelona vs Milan AC making her miss her preferred telenovella movies. The cost of effort can represent the psychological, financial and/or emotional load provided by the couple when accomplishing this effort. Clearly, greater effort is more expensive as it imposes a higher cost. It is appropriate then to say that the couple continuously provides efforts to achieve a greater well-being of the couple itself.

We adopt the following assumptions:

- the drift function 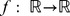 is continuously differentiable (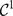),
- the well-being function 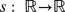 is 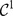-differentiable, non-decreasing, concave and saturated at 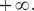,
- the cost function 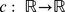 is twice continuously differentiable (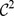), non-decreasing and strictly convex.

**Figure 1:**
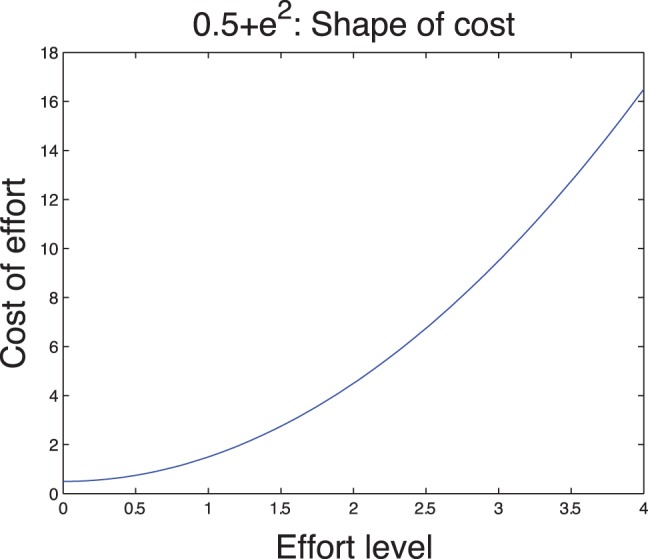
Typical shape of the cost function.

**Figure 2:**
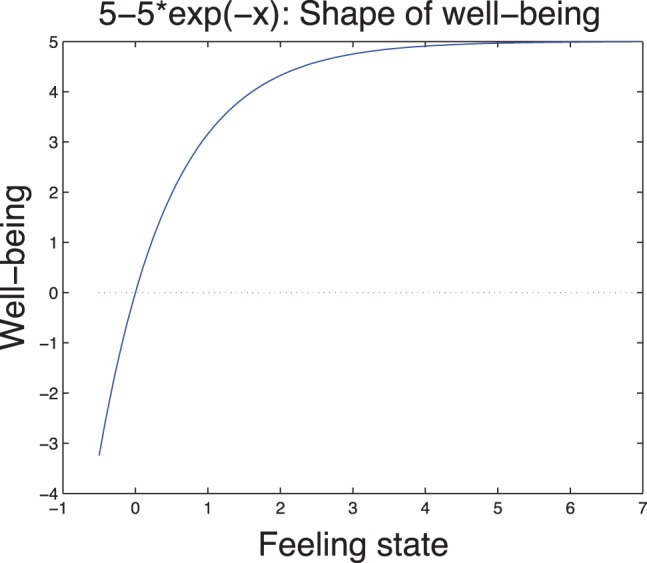
Typical shape of well being.

In Figures 1–2, we illustrate the typical well-being and cost functions.

In the next sections, we consider respectively an open-loop control problem, an optimal feedback strategy for the couple and a mean-field sentimental strategy.

## Results

### Optimal Open-loop Effort Control

A control law of the form 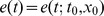 for 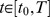, determined for a particular initial state value 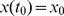 is called an open-loop effort. This does not depend explicitly on the state 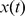. One seeks for an optimal open loop effort strategy by applying the maximum principle to the following problem.
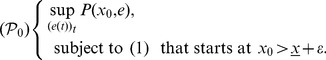
(3)


To do so, we use the Hamiltonian and the adjoint process. First we analyze the deterministic case (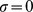) and then the open-loop noisy case.

**Deterministic Open-loop optimal effort.** When 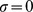, the Hamiltonian is
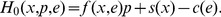
(4)


A maximizer of 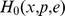 with the respect to the effort provides an open-loop optimal control associated with the co-state (adjoint) process 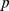.

The adjoint process of the optimal control is
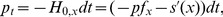
(5)with 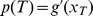. Note that 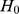 is strictly concave in 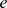. For concave function, the first order optimality condition is also a sufficient condition for interior point. The (interior) effort should satisfies 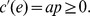 The positivity constraint of the effort suggests 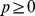 and then there is a unique solution.

The open-loop optimal control system via Pontryagin maximum principle [7] yields:
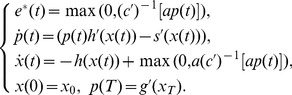
(6)


By differentiating 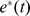 and combining with the above system, we arrive at
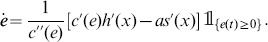
(7)


Hence, one gets the following dynamical system between the optimal control and the optimal feeling:
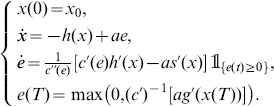
(8)


Our first question is about the well-posedness of the above system (8). Our goal is to provide a sufficiency condition for existence of a solution. We use fixed-point theorem to establish existence under the above assumptions. Since the functions 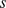 and 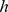 are 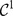-differentiable and the strictly convex function 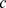 is 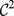-differentiable, (8) admits a local solution. Next we use a stochastic maximum principle approach to analyze the case where 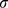 is nonzero, i.e., the stochastic case.

#### Stochastic optimal open-loop effort

Following the stochastic maximum principle technique, the adjoint processes for constant variance coefficient yields
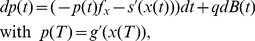
(9)where 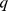 is the adjoint variable associated with the diffusion term. Let 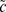 be the Legendre-Fenchel transform of 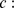




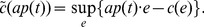
(10)It is well known that 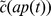 is convex in 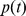. Furthermore, the optimal control is given by
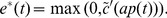
(11)


Using Ito’s calculus, the Euler-Lagrange system is given by
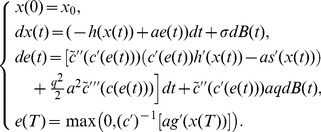
(12)


A sufficiency condition for existence of solution 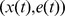 is the uniformly Lipschitz with respect to 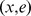 of the coefficient functions and uniformly Lipschitz condition for 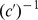 over the horizon 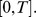


### Optimal Feedback Effort Strategy for the Couple

A feedback effort law is of the form 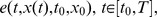 i.e., the effort depends on time 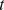, feeling 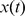 and possibly the initial conditions. Our motivation for feedback strategy comes from the following result from Jackson (1957), page 79: “*A family interaction is a closed information system in which the variation in outputs or behavior are feedback in order to correct the system’s response*”. This idea was re-used in the book [3] entitled “The Mathematics of Marriage”. The pay-off functional is written as
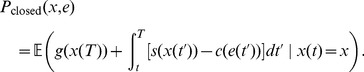
(13)


To find the optimal feedback control 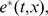 we use dynamic programming. Let 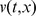 be the value starting from 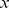 at time 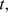 i.e.,
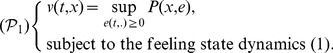
(14)


The Hamiltonian is given by
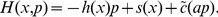
(15)


Since 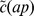 is convex 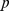 we deduce that 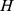 is convex in 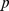. It is well known that, if there exists a twice continuous differentiable function 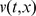 solution of
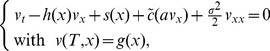
(16)then the stochastic optimal control effort is



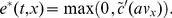
(17)Under the assumptions on 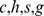 and given 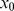 and the strictly concavity of the payoff function in 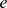 implies that there is a unique optimal control. Thus, the existence of the value follows.

### Mean Field Sentimental Games

There is a significant consensus around the idea that the society state (particularly the feeling states of the friends, parents, friends’ of friends and social environment) may influence the status of the couple. For example, if many of the friends are in lower feeling states, their divorce can be contagious. Moreover, a couple’s tendency to divorce depends not just on their friends’ divorce status, but also extends to their friend’s friends and so on. Thus we must consider the distribution of states within the entire society, i.e., the mean-field of states. Previous works neglected the influence of society and the couple’s network [4], [5]. In this paper, we take into consideration the mean-field state of the society in the sentimental dynamics of the couple. This leads to a *mean-field sentimental game*.

Roughly speaking, we introduce a society’s social influences (mean-field) and external shocks into the marital interaction model. To do this, we assume that the function 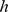 has the form 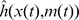 where 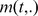 is the distribution of states of all couples in the society at time 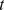. Based on the model (1), we state that the feeling state (feeling level) 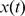 of the couple is modeled as a McKean-Vlasov Itô’s stochastic differential equation:
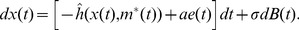
(18)where 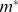 is the equilibrium distribution of states.

In this sequel the long-term payoff of a generic couple is written as
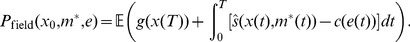
(19)


The objective of each generic couple is to maximize its long-term payoff through the mean-field fixed-point problem:
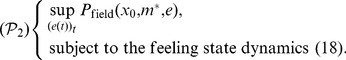
(20)


The happiness function 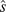 covers the well-being of the couple and the satisfaction of its network. It is natural that the happiness distribution over the network is concentrated in the family set. It is also known that the social network may contribute to enhance the well-being of the couple.

#### Mean-field sentimental equilibria

We define a mean-field sentimental equilibrium as a (Nash/Wardrop) equilibrium of the mean-field sentimental game. A mean-field sentimental equilibrium in feedback strategies is a situation in which no couple has incentive to move unilaterally from its effort feedback strategy.

Following [6], the mean-field equilibria are solution of the following backward-forward system for 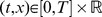 is
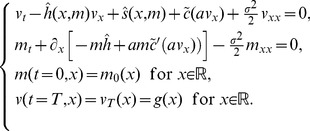
(21)


Under the above assumptions on 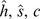 and the set of effort strategies, the mean-field sentimental game has an equilibrium.

**Result 1** *Asssume that the contribution of the mean-field to the feeling state of a generic couple is small. Then, the optimal level of effort for a long-term viability of the couple (during their lifetime) which keeps a happy relationship going is always greater than the effort level that would be chosen in a one-shot, i.e., 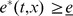, where 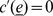.*

By assumption, the functions 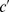 and 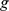 are non-decreasing, and the term 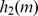 has only small influence in the drift. By small influence of 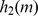 we mean that the uncontrolled system leads to a divorce or fluctuate feeling state if no effort is injected. The optimality equation (21) and the strict convexity of the cost 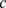 gives 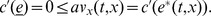 This means that 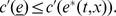 Using the inverse function 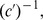 one gets 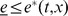 and there are several time 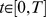 for which 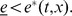.

#### Open-loop sentimental equilibria

In this section, we analyze the influence of the society state (mean field) on the couple. Besides, we assume that the functional 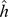, 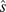 and 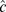 take on the following forms:
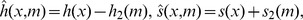
where 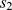 and 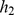 are smooth functionals of the mean-field distribution. Next we present two important results on the contagion of divorce.

**Result 2** *In a short horizon, Breaking Up is Hard to Do, Unless Everyone Else is Doing it Too.*

Consider a society where the majority of marriages are stable. This means that both 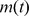 and 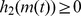 are high enough compared to 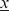 Thanks to the contribution of 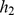 in the feeling state dynamics, the generic dynamics given in (18) has an higher value. The more the value of 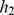 is, the more the feeling state will be. This means that 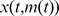 will go to a higher state than the case without mean field. Thus, Breaking Up is Hard to Do in a short horizon even if there is no effort from the couple.

**Remark 3 (Societal benefit!)** *If 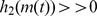 then the marriage remains maintained over time even if the couple effort is minimal. This is because the mean-field contribution plays the role of a positive effort. The network of the couple is having a big positive influence on their feeling state.*

**Result 3** *If the mean-field has a tendency to the divorce states then, there is a contagious phenomenon for divorce, i.e., starting from 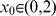 the couple state will degrade due to the influence of the mean-field toward a negative feeling state. In particular, Breaking Up is not seen as a negative thing in that society because the majority is Doing it Too. Furthermore, stabilizing a marriage will require more effort, and hence it will be more expensive.*

Let 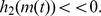 The mean-field is concentrated to negative values below the divorce threshold range. The majority has a tendency to the divorce. Thus, the feeling state of a generic couple goes towards to negative values if no effort is made. A high effort is required to bring back and maintain the feeling state to a satisfactory one. The threshold effort 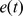 to balance is 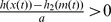 which requires some time and cost. In this configuration, a divorce is not seen a negative thing by the society because the majority of the society is in a divorced state. The negative term from the society will required more effort (and hence more cost) for the stabilization of a marriage.

## Social Welfare

In this section we aim to maximize the social welfare. It consists to solve the following mean-field control problem:
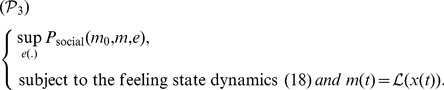
(22)where 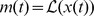 is the distribution of 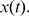 Note that the social welfare problem 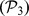 is different than 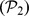 because now a control action 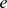 affects immediately the distribution 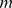




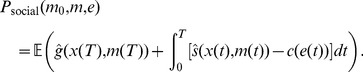
(23)Define the function 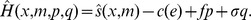 The stochastic maximum principle of mean-field type yields
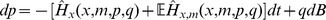
where 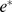 is the maximizer of 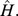 Note that the impact of the society on the functions 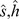 and 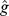 one gets the same optimality equations as above. However, the presence of the mean-field term 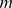 changes drastically the behavior of the adjoint process 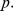


## Discussions: Illustrative Model of Marriage

In this section we present a mathematical model of marriage for a specific functional 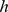 in (1). In fact, we study the dynamics introduced in the “Mathematics of marriage” book [3] in which we take into consideration a control term 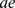 and a noise term. The dynamical system in the case where
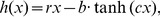
(24)is written as
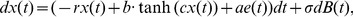
(25)with 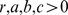. The choice of the function 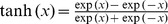 in (24) is widely justified in the “Mathematics of marriage” [3] as well as in evolutionary game theory [1] as a resulting from the imitative logit dynamics (see [2]). The model is widely supported by many psychologist and sociologist authors (see [3]). Note that the function 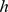 defined at (24) does not belong to the class of functions studied in [4].

We choose 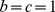. Then, the sentimental dynamics with noise becomes
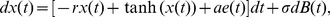
(26)where the effort 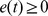 is a control variable. The parameter 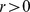 refers to the type of the society where the couple lives. We also perform numerical examples to illustrate the effectiveness of the theoretical results. To do this, let consider the mathematical model (1) with 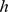 defined in (24). The cost and the well-being functionals are chosen as



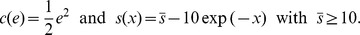



It is worth noticing that the above choices of 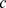 and 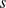 ensure the existence of solutions for the problems (8), (12) (17) and (21).

### Steady States of the Sentimental Dynamics

According to the type of the society, we analyze the uncontrolled (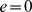) sentimental dynamics.

**Figure 3:**
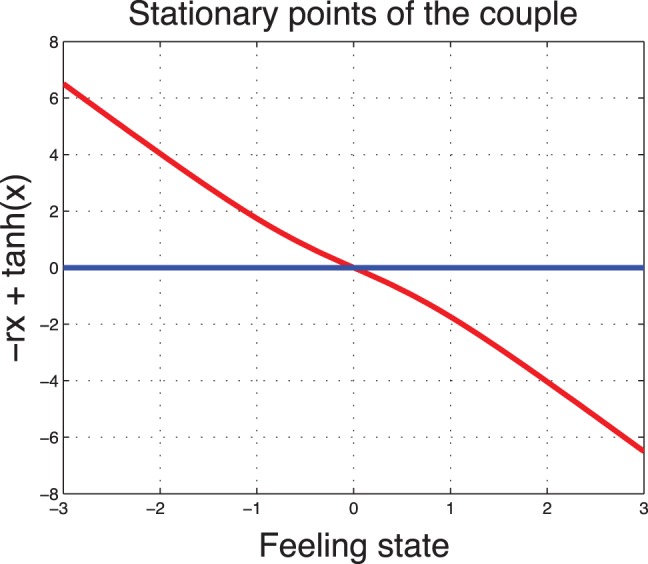
Steady states of the uncontrolled (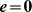) dynamical system: high type society 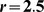 (one steady state).

**In a high type society (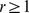).** For 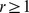 the uncontrolled system (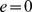) converges to zero independently of the starting point. This can be interpreted as if the couple feeling state will degrade over time if there is no effort. The uncontrolled feeling state has only one steady state which is illustrated in Figure 3.

**Figure 4:**
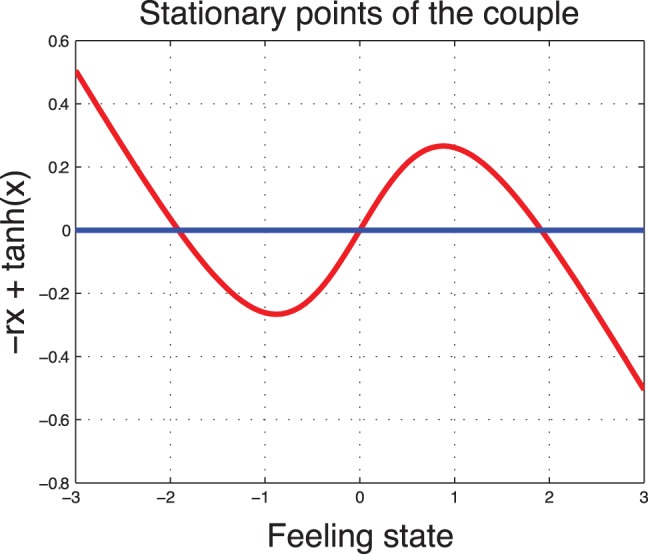
Steady states of the uncontrolled (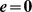) dynamical system: low type society 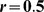 (three steady states).

**In a low type society (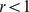).** The degradation rate is less aggregative. We observe that in a society of low type, the uncontrolled system (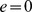) has three steady states. Two of them are stable and one is unstable. The unstable feeling state is the state 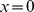 because the derivative at 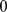 is 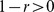. The another two steady states are symmetric with respect to the origin. The one positive can be seen as if the marriage stays for life. A negative steady state characterizes a divorce situation. The two non-zero states are stable. Indeed, the derivative at the steady points can be written as 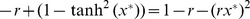 which is negative when 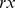 is near 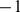 or 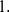 In figure 4 we illustrate the zeros of the drift function 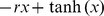 for different values of the societal parameter 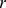.

### Evolution of Uncontrolled Feeling States

Here, we show the societal influence on the sentimental dynamics of a couple without effort (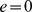). In this case, we observe the variation in time of the feeling state for the two types of society. Numerical experiments for the deterministic (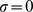) evolution and stochastic (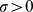) dynamics in high type society (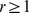) and in low type society (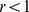) have been performed.

**Figure 5:**
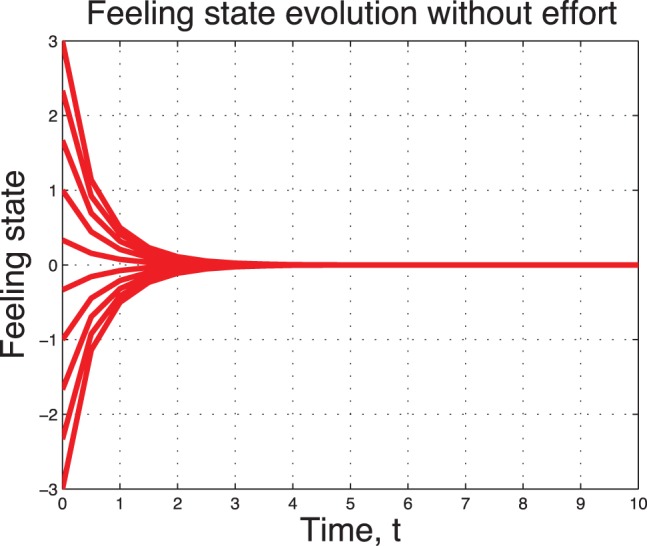
Uncontrolled sentimental dynamics without noise for 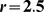.

**Figure 6:**
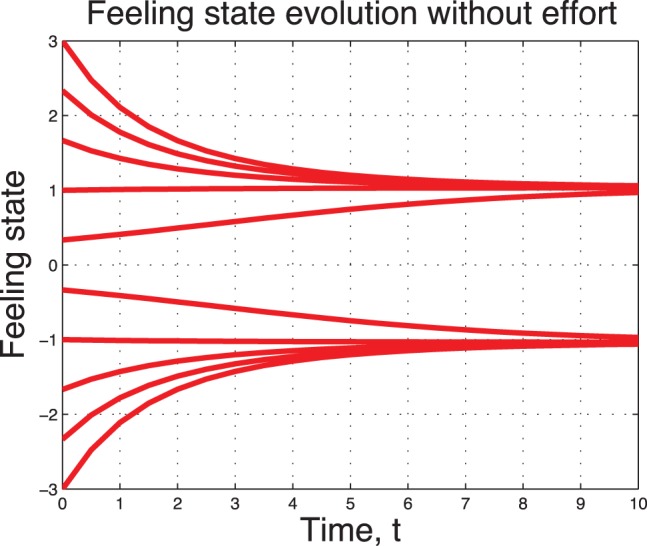
Uncontrolled sentimental dynamics without noise for 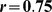.

A sample of 10 non-working couples with different initial feeling states are depicted in figure 5 and in figure 6. We observe a fast convergence to the state 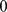 in the high type case 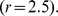 In the low type case 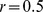, we observe three steady states. Two (one positive, one negative) of them are stable states.

### Stochastic Evolution

Now we introduce a noise to better capture the fluctuations observed in real life. We observe that with a small variance 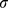 the feeling state of the couple leads to a fluctuating trajectory around the deterministic sentimental dynamics. However, a bigger noise leads to two main branches and a non-zero probability to switch from one branch to another.

**Figure 7:**
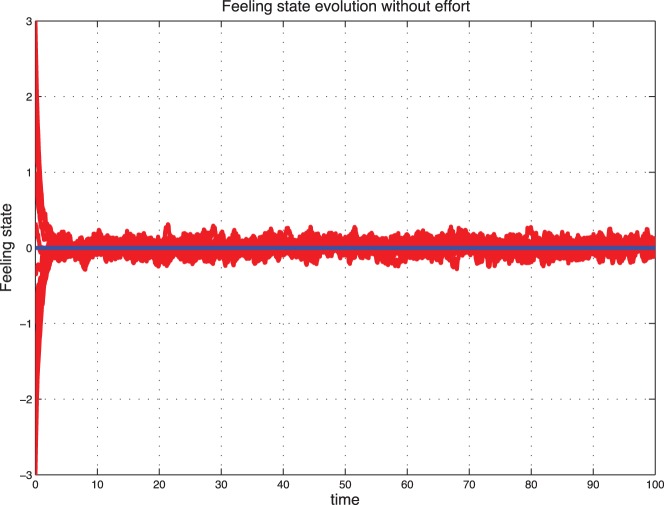
Noisy sentimental dynamics without control for 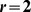.

**Figure 8:**
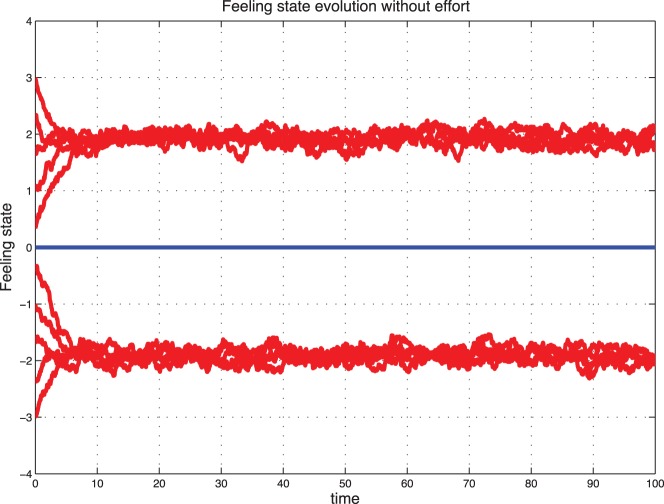
Noisy sentimental dynamics without control for 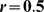.

Figure 7 represents the stochastic evolution of sentimental dynamics without control for 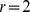. Figure 8 represents the noisy evolution of feeling states for low type 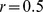.

### Sentimental Dynamics with Effort

**Figure 9:**
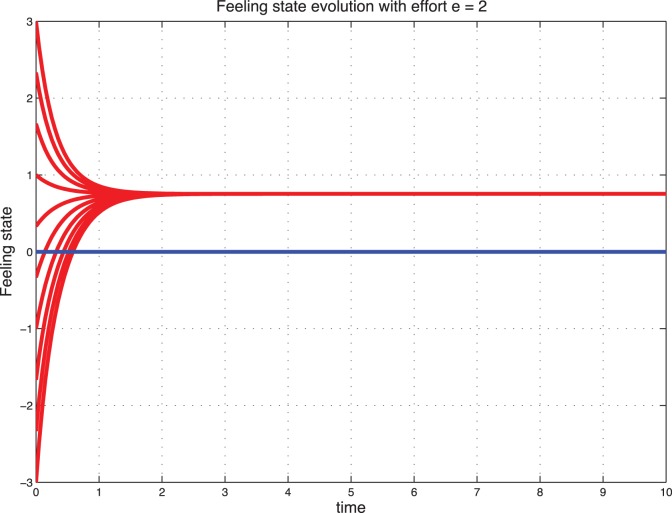
Deterministic sentimental dynamics with control for 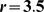.

**Figure 10:**
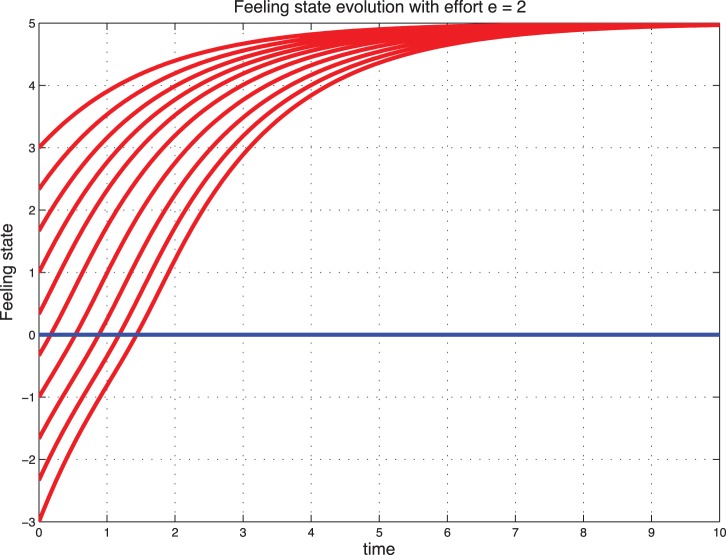
Deterministic sentimental dynamics with control for 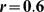.

**Figure 11:**
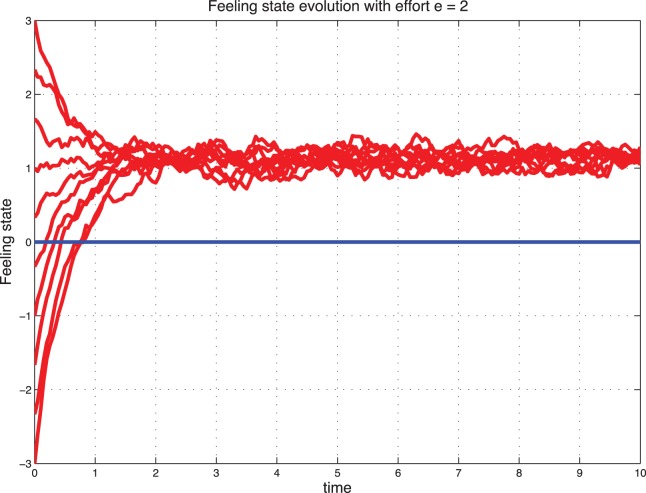
Stochastic feeling state with control for 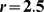.

**Figure 12:**
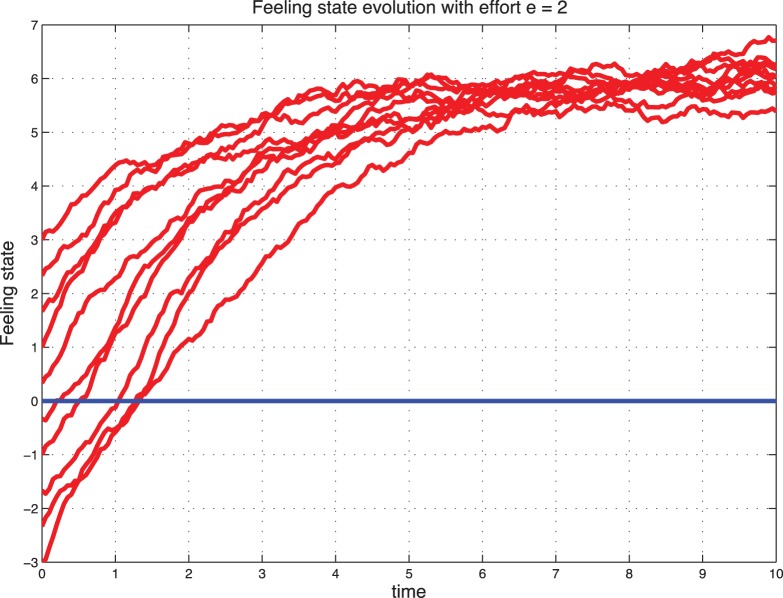
Stochastic feeling state with control for 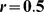.

Interestingly, when we introduce the effort, a catastrophic situation (starting from 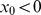) can be reconstructed with effort and stabilize to a positive feeling state. These cases are illustrated in Figures 9 and 10 for the deterministic dynamics and in Figures 11–12 for the stochastic dynamics. Thus, the effort plays an important role in maintaining the marriage or the cohabitation. On the other hand, high effort may be costly to the couple. Thus, it is crucial to find an acceptable tradeoff.

**Figure 13:**
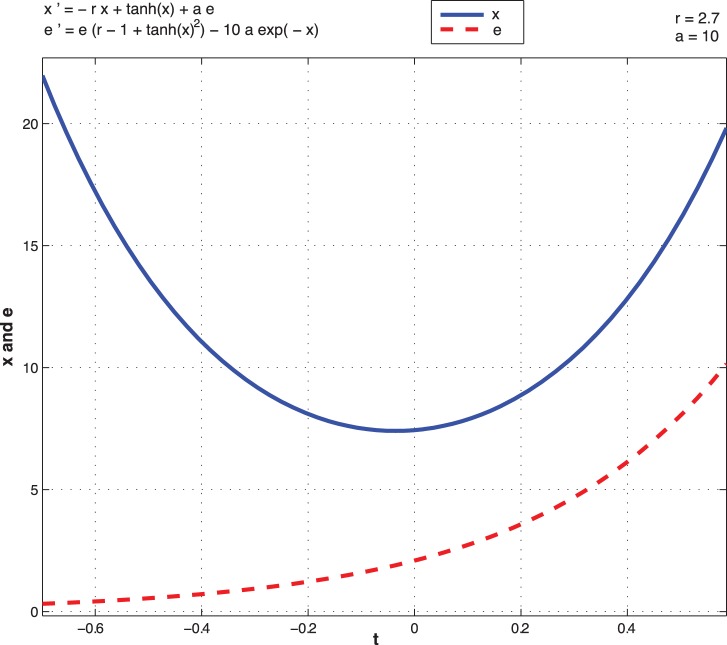
Open-loop optimal control system with high type 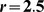 as a function of time.

**Figure 14:**
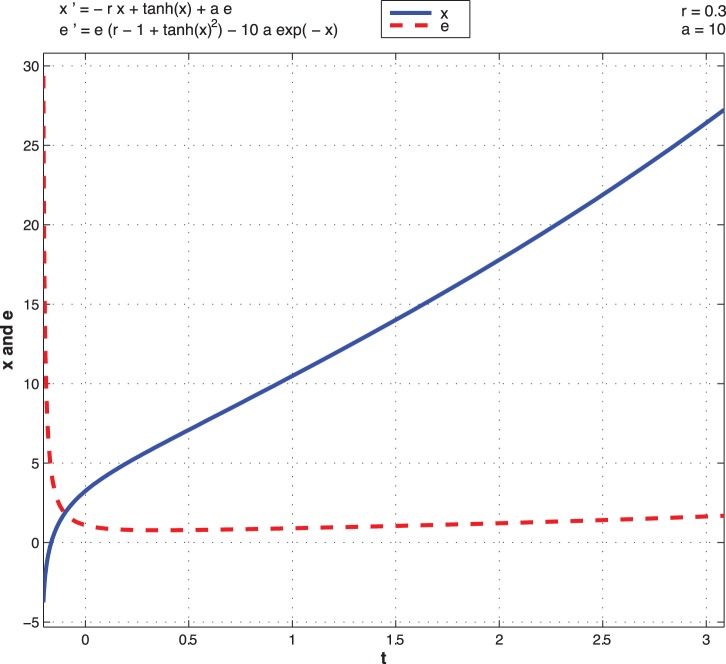
Open-loop optimal control system with low type 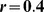 as a function of time.

Next we depict the dynamics driven by optimal control as a function of time. From the open optimal control of the non-noisy dynamics (8), we observe two different situations. Figures 13 and 14 represent the trajectories of 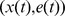 for different initial conditions for both high and low type for 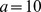.

**Figure 15:**
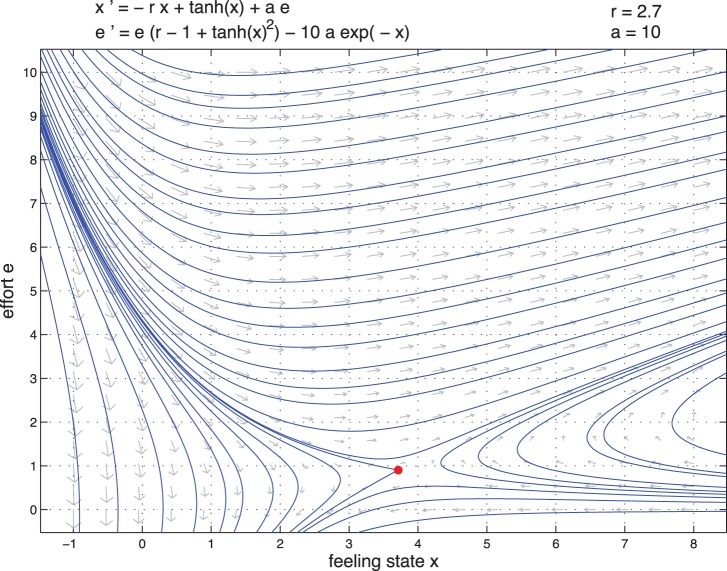
Vector field state versus effort for high type 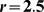.

**Figure 16:**
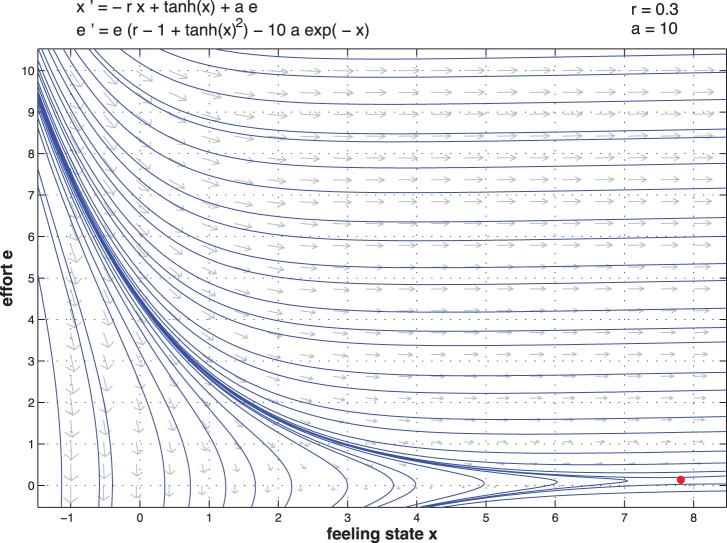
Vector field state versus effort for low type 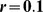.

Figure 15 and Figure 16 represent the vector field between feeling state and effort of the couple for high and low type. We observe there is an invariant set which can maintain very high effort and high feeling state.

### Stochastic Optimal Control

**Figure 17:**
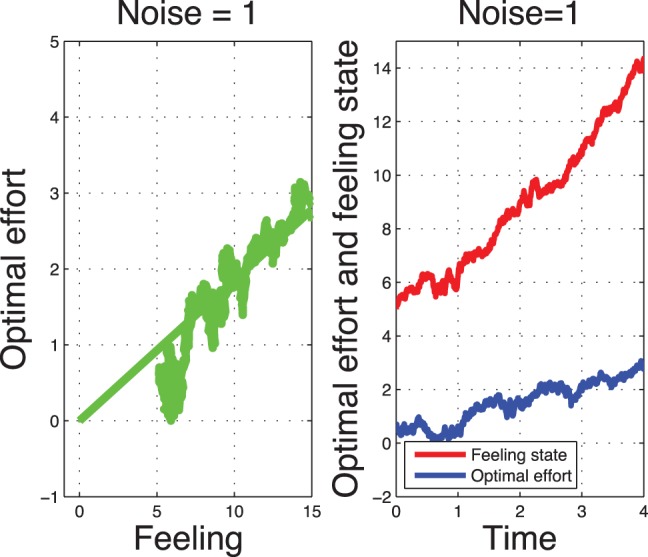
Open-loop stochastic optimal control system for a low type.

Figure 17 depicts the stochastic optimal control trajectories and optimal effort. We observe that a small noise in the feeling state may have big consequences in the optimal effort (and the cost of effort).

### Dynamic of the Optimal Feedback Effort

The experiment consists in observing a couple over a finite horizon. We suppose that the couple finishes the horizon 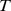 with the same heritage of feeling state whatever the initial feeling state is. In other terms, the functional 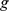 is constant, therefore the terminal optimal effort is zero. To describe the dynamics of the optimal feedback strategy of such a couple, we applied an implicit (time) backward scheme to the backward differential equation (16). The Hamiltonian is approximated using Lax-Friedrichs approach to preserve monotonicity, consistency and continuity properties. Two numerical experiments are performed with respect to the type of the society.

**Figure 18:**
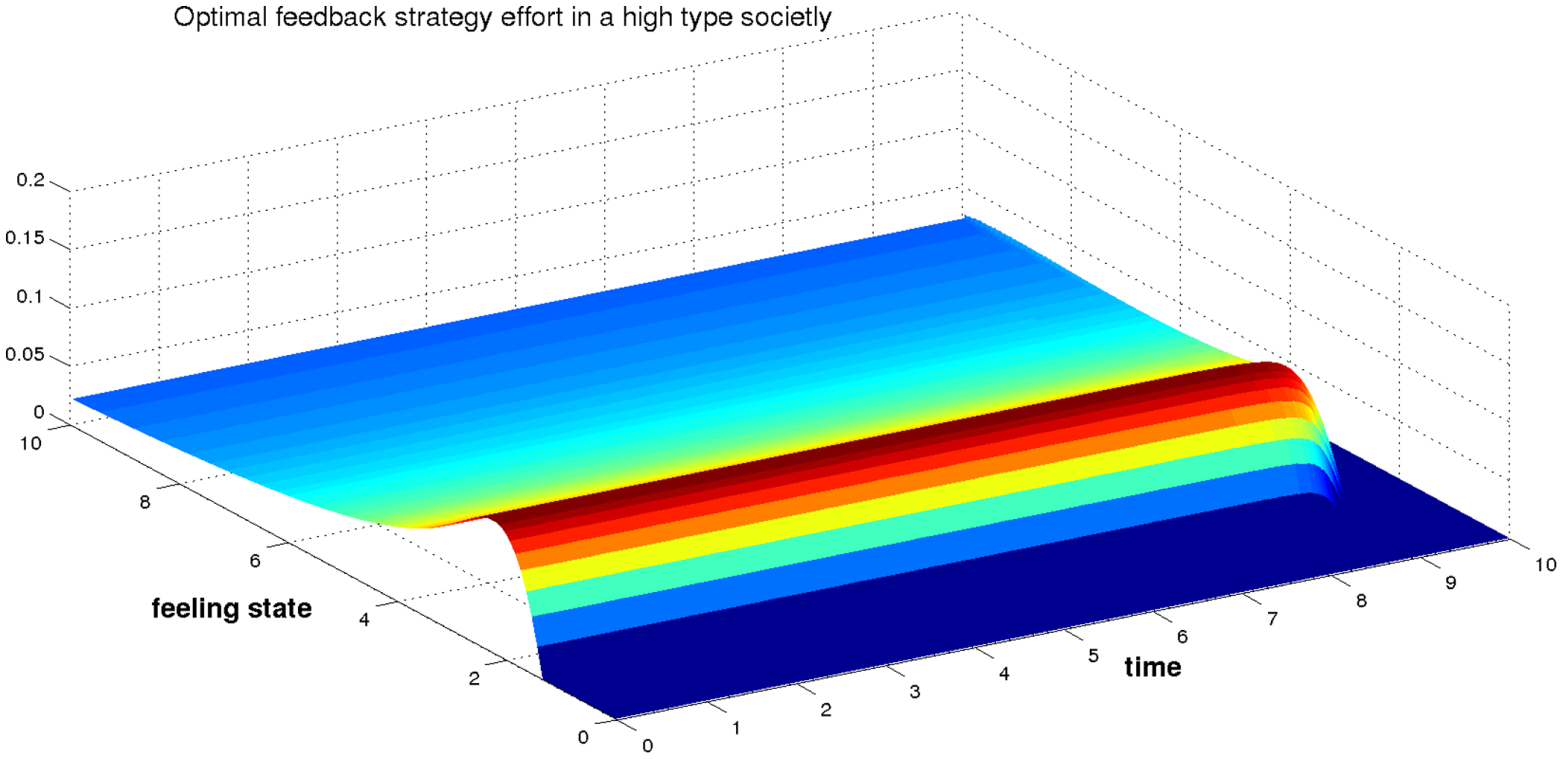
The optimal strategy provided by a couple in a high type society 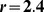.

The couple starting with a low feeling state is to provide no effort. For the conjoints of such couple, it is hard to resist due to the influence of the high type society. From Figure 18, one can see that for a couple starting with a fairly good feeling state (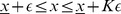) the optimal strategy is to do actions. Indeed, the couple will break up because of the negative influence of society and will line up with a null optimal effort. Also, we observe the same phenomenon for a couple starting with a higher feeling state. The optimal effort is nevertheless important because starting at a higher feeling state can counterbalance the negative influence of the society.

**Figure 19:**
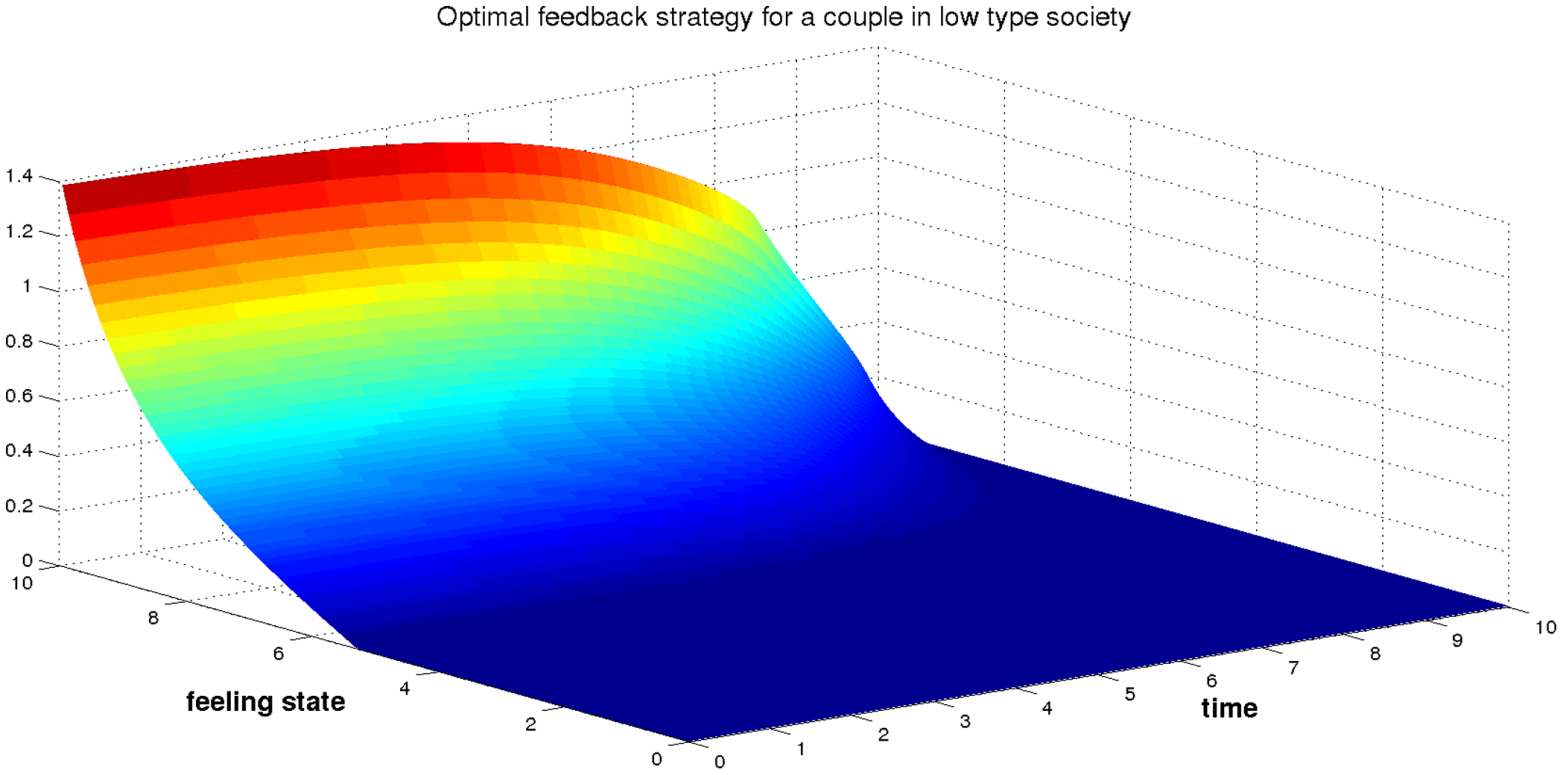
The optimal effort strategy of a couple in a low type society 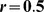.

The optimal strategy for a couple starting with low feeling state is to provide zero effort. This is not surprising since in a low type society living, a couple follows a societal phenomenon. We can see from Figure 19 that the optimal strategy effort for a couple starting with high feeling state is a time-decreasing function. In that case, doing good action is motivated more by seeking ideal happiness than by up-holding the couple. Overall, we observe that the optimal strategy is cheaper in a low type society than in a high type society.

### Mean Field Equilibrium Trajectories

We now address the numerical simulations for the mean-field equilibrium (21). The functional 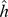 and 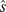 are chosen as defined in (22), the functional 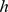 is specified in (24), the effort cost and the well being intrinsic to the couple are given by (27).

The numerical experiment consists here in observing the evolution of the distribution 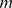 representing the society in which a given couple lives. For the sake of simplicity, we suppose that the reference couple has an almost constant optimal effort in a particular window. This implies that the value 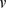 is linearized with respect to 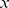 and constant in time. The numerical task is then to compute the Fokker-Planck-Kolmogorov equation in (21). The developed algorithm focuses on unnormalized distribution (positive measure).

**Figure 20:**
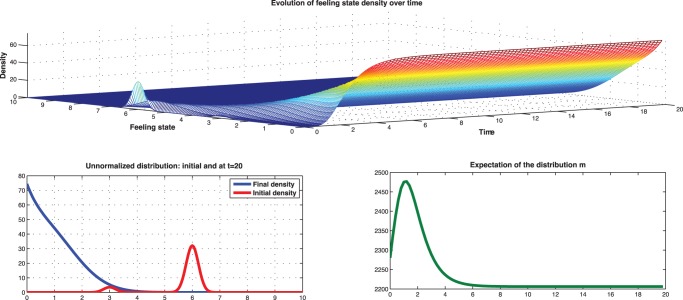
Low type: Evolution of mean-field starting from Gaussian concentrated at 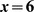 with standard deviation 1.5.

Figure 20 represents the evolution of the mean-field equilibrium with initial Gaussian distribution centered at 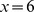 with standard deviation 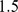 for a low type.

**Figure 21:**
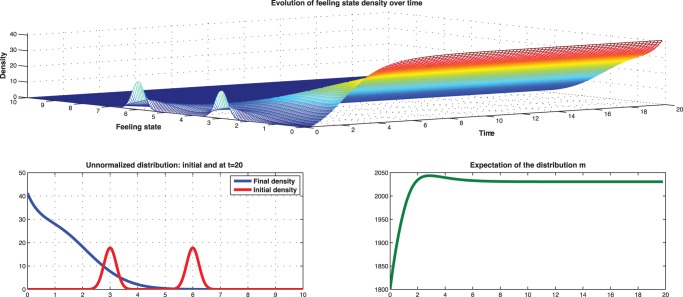
Low type: Evolution of mean-field starting from mixture Gaussian concentrated at 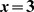 and 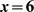 with standard deviation 1.5.

When the initial distribution is a mixture of Gaussian distributions centered at 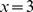 and 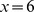 with standard deviation 1.5, we observe in Figure 21 that the state distribution will propagate rapidly to be concentrated at the extreme in a relatively short time.

**Figure 22:**
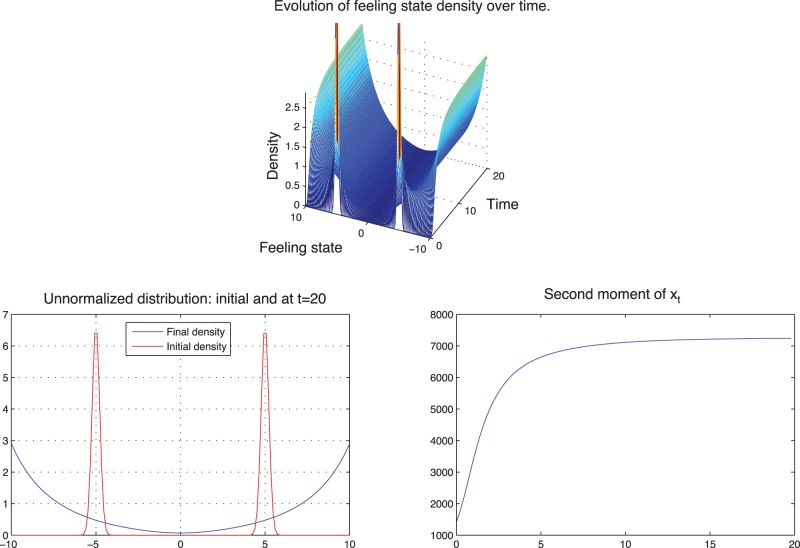
Mean-field equilibrium: the unnormalized distribution is concentrated at the two extreme boundaries.

For the mean-field equilibrium, the symmetric steady states observed previously in the case where the control was a constant, are not steady state anymore because the feedback control 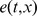 is now dynamic in state as time goes. We observe that if the initial mean-field is a mixture of Gaussians centered at −5 and at +5 with standard deviation 0.05 then the feeling state will propagate and will be concentrated at the two extremes at the final time (Figure 22).

## Conclusion

In this paper we have proposed a mean-field game model for marriage, cohabitation, divorce and remarriage. Our study suggests that the optimal effort may help in sustaining marriage and cohabitation if the cost of that effort is acceptable and the initial couple state is not too low. This helps us to understand at least theoretically the key processes related to marital dissolution, co-habitation separation, stability and fluctuation. The study can be useful to design or evaluate an adequate intervention. It also suggests that knowing many divorced people may influence the status of a marriage. The more divorced people you know, the riskier your own marriage. However, a marriage doesn’t break down just because friends are divorcing. Marital breakdown depends on many factors including effort and mean field.
